# Autoimmune Health Crisis: An Inclusive Approach to Addressing Disparities in Women in the United States

**DOI:** 10.3390/ijerph21101339

**Published:** 2024-10-10

**Authors:** Syreen Goulmamine, Sarah Chew, Irene O. Aninye

**Affiliations:** Society for Women’s Health Research, Washington, DC 20036, USA; syreen@swhr.org (S.G.); sarah@swhr.org (S.C.)

**Keywords:** autoimmune disease, type 1 diabetes mellitus, Hashimoto’s thyroiditis, health disparities, health equity, intersectionality, systemic lupus erythematosus, women’s health

## Abstract

Autoimmune diseases are identified by the chronic inflammation and tissue damage resulting from unregulated immune responses throughout the body. Systemic lupus erythematosus, type 1 diabetes mellitus, and Hashimoto’s thyroiditis are among the 80+ characterized conditions, 80% of which are diagnosed in women. The compounded effects of biological sex and hormones; social identities, such as age, race, and gender; and other determinants on health highlight a pressing need for an inclusive approach to address disparities for women living with autoimmune diseases. Such an approach must recognize and incorporate intersectional experiences of diverse populations of women into biomedical research, clinical practice, and policy solutions. Research must prioritize inclusive designs, data collection, and representation of women in clinical studies. Clinical care must focus on developing guidelines and promoting patient–provider interactions that meet a range of demographic needs. Health care policies must support financial investments in research and equitable access to care. This review provides an overview of the impacts of autoimmune diseases on women’s health through an intersectional lens, identifies persistent gaps in addressing the unique needs of women, and proposes recommendations for a comprehensive, equity-focused approach to mitigate disparities and better serve all women at risk for or living with autoimmune diseases.

## 1. Introduction

### 1.1. Autoimmune Diseases in Women’s Health

Autoimmune diseases are an emerging public health crisis in the United States due to their rising prevalence and increasing health care costs, impacting up to 8% of the population. These diseases occur when the immune system mistakes the body’s own healthy tissue for harmful antigens, resulting in chronic inflammation and tissue damage. There are demonstrated sex and gender differences in autoimmune disease prevalence, with more than 80 types of conditions predominately affecting women compared to men. Approximately 80% of all patients diagnosed with autoimmune diseases are women [1,2].

Systemic lupus erythematosus (lupus) is characterized by widespread inflammation and multisystem organ dysfunction—most commonly in the joints, skin, kidney, brain, and blood vessels. Lupus has a 9:1 predominance in women and is three to four times more prevalent in Black and Hispanic females than White females [3,4]. Patients often experience fatigue, joint swelling and pain, and a characteristic butterfly-shaped rash on the cheeks and nose. Even individuals with well-managed lupus can experience flares in between extended periods of low disease activity. The condition can be triggered by estrogen hormone levels, stress, environmental exposures, and/or certain medication, requiring constant vigilance to manage this chronic health condition. Furthermore, the systemic dysfunction can result in misdiagnosis for other conditions, as well as complications and multi-morbidities throughout the patient journey [5].

Thyroid autoimmunity is often considered a gateway to other autoimmune states because individuals diagnosed with more than one autoimmune disease are likely to have a thyroid autoimmune disease—either presenting clinically first or subclinical post-diagnosis [6,7]. One type of thyroid autoimmune disease, Hashimoto’s thyroiditis, attacks the hormone-producing cells of the thyroid gland, resulting in hypothyroid activity. The risk of developing Hashimoto’s is four times higher for adult women than men and is most common in middle-aged women [8]. Hashimoto’s presents with notable complications that can impact metabolism and weight gain, cardiovascular health, and pregnancy outcomes. Studies suspect that the increased incidence of postpartum autoimmune thyroid disease might be due to the body’s hormonal and immunosuppression reset from pregnancy, with up to 5% of all women developing thyroiditis after pregnancy [9].

Although certain autoimmune diseases have similar or slightly higher prevalence in men, disparities in sequelae can still be observed in women. For example, type 1 diabetes mellitus (T1D) poses a greater risk of excessive weight gain in girls during puberty that can exacerbate an insulin resistance resulting from the prior destruction of insulin-producing cells in the pancreas [6]. T1D was historically referred to as juvenile diabetes because of its early age at presentation and diagnosis; however, it is a condition that can also be diagnosed in adulthood. T1D can have lifelong impacts; remission, if attained, is usually temporary. In fact, girls with T1D are at increased risk of hyperandrogenism and polycystic ovary syndrome, with future complications that include infertility, cardiovascular disease, and end stage renal disease [10,11]. T1D’s global incidence of 15 per 100,000 population has been increasing 3–4% annually over the last 30 years, with direct costs estimated at well over USD 100 billion per year in the United States [2,12]. While genetic and environmental factors, such as chemical pollutants, and dietary changes, personal lifestyle, stress, and obesity have been linked to T1D, the cadre of evidence is still premature to solve this global grand challenge.

Autoimmune diseases can affect almost any tissue of the body—most often a women’s body—yet the commitment to elucidate the role of sex and hormones in these diseases is subpar. In addition to these biological influences of autoimmune diseases, sociocultural factors can significantly contribute to the disparities observed in women’s autoimmune health. Environmental exposures and triggers, disease progression due to delayed diagnosis, access to quality treatment options and education, and financial stability are recurring determinants that impact women’s health. Examining the intersecting identities and influences on a woman’s health can provide better insights into the broader patterns and mechanisms of autoimmune diseases, potentially leading to more personalized and effective treatment approaches.

### 1.2. Understanding Intersectionality in Women’s Health Care

An inclusive approach to addressing disparities in women’s health requires recognition of the overlapping factors of gender, race, socioeconomic status, and other identities that influence health outcomes. Often referred to as intersectionality within biomedical research and public health, this theory was described in 1989 by civil rights advocate and law professor Kimberlé Crenshaw and originated outside of the medical field. Crenshaw’s initial analysis considered the intersection of race and sex (and gender) in the legal field, to illuminate how multiple forms of inequality or disadvantage can compound themselves and create obstacles that often are not understood within conventional ways of thinking of anti-racist politics or feminist theory [13]. Other scholars have expanded her definition to consider other structures that may form systems of inequity and disadvantage to an individual, including age, disability, and socioeconomic status.

Olena Hankivsky, for example, introduced an intersectionality-based framework to support public health and policy actors in understanding how inequities in health outcomes are influenced by the multi-dimensional contributions of social location, power relations, and individual experiences [14]. Hankivsky’s definition has been utilized globally to understand the many systems and identities impacting an individual’s health and wellness, including by the U.S. Department of Health and Human Services [15,16,17]. Intersectionality has become a valuable approach to analyze social structures and historical frameworks in biomedicine that have disregarded diverse perspectives and marginalized groups and contributed to inequities and disparities in human health and disease. As such, efforts to promote health equity must consider these intersecting factors to effectively address the unique needs of marginalized populations.

Autoimmunity is impacted by a variety of factors, including genetics, lifestyle, environment, and access to resources. Identities and structures within society and the health care ecosystem have different—and often disparate—impacts for women or certain subgroups of women, requiring an intersectional lens to better understand these disparities and to improve the diagnosis, treatment, and quality of life for women with autoimmune diseases. Intersectionality has utility to promote equity throughout the health care continuum—from the investigation of disease etiology and development of therapeutics to the delivery of health care services and legislation that supports accountable sustainability. Furthermore, when considering key players in the health care ecosystem that have a shared responsibility to improve autoimmune disease patient outcomes, three jurisdictions consistently emerge: research, clinical care, and policy. Successful health care reform relies on this collaborative three-pronged approach, and sustainable change must prioritize the inclusion of all impacted populations.

Based on intersectional theory frameworks, this review introduces an approach to address autoimmune disease disparities in women that considers how variables (identities and structures) relevant to these chronic conditions individually and interconnectedly impact the experiences and outcomes for this under-served, yet rapidly growing, demographic in the United States. The three autoimmune diseases highlighted throughout this review were selected to illustrate the scope of autoimmune disease burden in women—Hashimoto’s disease has widespread prevalence in women; lupus occurs with significant disproportionality in women compared to men; and T1D has similar prevalence in women and men, but has disparities in morbidity and sequelae experienced by women (Table 1).

Articles identified for this review were initially identified through database and academic search engine queries of PubMed, Web of Science, and Google Scholar. Key search terms included autoimmune disease, Hashimoto’s thyroiditis, health disparities, intersectionality, systemic lupus erythematosus, type 1 diabetes mellitus, and other relevant public health and health care policy terminology. All retrieved articles were manually reviewed for relevance to ensure the quality of the sources. Focusing on publication dates from 2015 to present, original research studies, systematic reviews, reports, and seminal papers were selected for this review.

## 2. Equity and Inclusion in Research Study Design

Women and individuals from racial and ethnic minority groups have been historically excluded from medical research, resulting in gaps in knowledge and clinical strategies to address health and disease for these populations [27]. The gender gap in autoimmunity is undeniable and becomes particularly evident surrounding the three major hormonal transitions in a woman’s life course—puberty, pregnancy, and menopause [6]. Certain autoimmune diseases demonstrate notable prevalence and disease severity among different racial groups, such as lupus in African Americans and autoimmune hyperparathyroidism among Native Americans, and celiac disease among Caucasians [28]. Thus, research questions and study designs in the autoimmune space must bring to the forefront how race and ethnicity, sex and gender, and transitional life stages contribute to disease development and care, particularly for women.

### 2.1. Race and Ethnicity

Race and ethnicity are recognized constructs and structures relevant to human studies; nevertheless, in health research, ethnic and racial groups are often excluded or incorrectly classified, leading to significant gaps in understanding and addressing the unique health needs of many populations. Moreover, the nuanced differences and heterogeneity within ethnic and racial groups, such as Southeast Asia vs. South Asia, the diverse identities within the Latin community, or the distinction between African, Black, and African American are often not adequately represented or measured [29]. Brigham and Women’s Hospital examined two decades of data from over 20,000 clinical trials and found that less than half of the trials reported race and ethnicity data [30]. These oversights hinder the development of effective health interventions and policies, as they fail to account for the diverse experiences and challenges faced by these groups, ultimately impacting the quality of women’s health care.

Health-related research studies, including clinical trials, in which race or ethnicity are variables in the study design, typically use the five race categories (American Indian or Alaska Native, Asian, Black or African American, Native Hawaiian or Other Pacific Islander, and White) and two ethnicity categories (Hispanic or Latino) outlined by the Office of Management and Budget’s Statistical Policy Directive, which is also used for all federal reporting. This standard’s limited options may not accurately represent the diversity within a study population, hindering disaggregation of data or creating unactionable data gaps. For example, almost 50 years ago, a global study showed that estrogen levels were higher in Asian (Japanese) and African (Bantu) women than in Caucasian (Canadian) women [31]. Lupus, which is more prevalent among African American and Asian women, has been associated with antibody-mediated Th2 immune signaling—a response that is favored by high levels of estrogen hormone [6]. Stratification of race data within the Asian and African communities would allow researchers to more accurately assess the relationship between estrogen levels, autoimmunity, and disease incidence among each subpopulation of women—rendering a greater overall understanding of lupus disparities for women.

In March 2024, an updated directive (Directive No. 15) established a new race category for Middle Eastern and North African individuals, distinguishing them from the White category. When evaluating immunological parameters for lupus, Arabs had higher prevalences of almost all autoantibodies when compared to Europeans [32]. The previous misclassification of this population into other racial/ethnic categories or, often, the altogether omission of this group from clinical data collection leads to clear gaps in addressing their autoimmune disease and other health needs [33]. Directive No. 15 also called for the collection of additional details beyond the required minimum race and ethnicity categories to allow for further disaggregation in collection, analysis, and presentation of data where useful and appropriate. This directive is a start for expanding opportunities for study participants to select from more intentional race and ethnicity instruments. Hopefully, it will provoke researchers and other data-collecting actors to promote more accurate data representation, flexibility in data analysis, and cultural sensitivity, as well as to expose racial and ethnic disparities that may have been previously hidden within broader categorizations, especially among women with autoimmune diseases.

### 2.2. Gender (and Sex)

Researchers who work with humans should consider how sex and gender affect their subjects and incorporate it in the study design as applicable. The National Academies of Sciences, Engineering, and Medicine define gender as a social and cultural variable that encompasses four key domains—identity and expression, roles and norms, power relations, and equality and equity—typically associated with certain sex traits [34]. Often conflated with sex (a construct based on a cluster of anatomical and physiological traits, genetics, and hormones), gender can significantly influence health outcomes, but is rarely included in studies related to immune responses [35,36]. Sex and gender are considered independent factors that impact health and thus necessitate data collection as separate variables.

Gender norms and societal expectations can influence the diagnosis and treatment of autoimmune diseases. The dismissal of women’s symptoms as psychosomatic or ‘just in their heads’ has contributed to documented delays in diagnosis for many autoimmune diseases, including lupus and Hashimoto’s thyroiditis [37,38]. Gender-based roles for women, such as caregiver, are associated with increased stress and sequelae. With an estimated 61% of family caregivers identifying as women, various studies have highlighted caregiving-related stress as a hallmark symptom of this role [39]. Gender can also interact with genetics in the development and outcomes of immune responses [40]. The literature further suggests that physical and psychological stressors can serve as potential epigenetic modulators in the development of autoimmune disease [41,42]. As such, the epigenetic impacts and potential outcomes on autoimmunity that result from gender-based societal pressures and stressors are yet to be fully understood.

Gender identity can be particularly challenging to incorporate into a study because it is self-identified and may change throughout an individual’s life course. However, concerted effort must be made to attend to this variable, as it has far-reaching and nontraditional implications in human health. For example, populations that may be impacted by not distinguishing gender and sex as unique variables in women’s health include those who identify as non-binary or transgender men. These individuals may carry genetic risk factors or have hormone levels traditionally associated with women, thus warranting attention within the women’s health research community. This is especially important given a high density of immune-related genes are located on the X chromosome, with relationships identified to specific sex-related diseases [40]. It is critical to recognize the diversity within gender identity, as these individuals offer another layer of intersectional considerations unique to their identity that may impact autoimmunity and health.

### 2.3. Life Stage

Women experience significant endocrine changes during reproductive life stages, namely puberty, pregnancy, and menopause [6]. These hormonal shifts have profound effects on the immune system and can alter women’s susceptibility to develop an autoimmune disease. Animal and human studies have showcased the relationship between endocrine transitionary states in women and the etiology of specific autoimmune diseases, including T1D and lupus [6,43,44].

During puberty, T1D has been shown to have a greater risk of complications, such as excessive weight gain and comorbidity with polycystic ovary syndrome [10]. Autoimmune disease can also complicate pregnancy, adding to any pre-existing immunologic challenges or leading to the development of another autoimmune or gestational disease. For example, Hashimoto’s thyroiditis can present for the first time during pregnancy; however, the incidence of disease onset is especially increased postpartum and can occur with a postpartum mood disorder [43,45]. During the menopause transition, female patients with T1D have a higher mortality risk compared to male patients [46]. Researchers must dedicate efforts to investigate the role of life-stage endocrine transitions in autoimmune diseases, in order to aid in prediction, prevention, and treatment of women throughout the lifespan [47].

## 3. Enhanced Clinical Care Encounters

Health disparities in autoimmune diseases are exacerbated in clinical settings that do not incorporate social determinants and patient-centered approaches to clinical practice guideline development and delivery of care, given that nonmedical factors can directly and indirectly impact health outcomes [48]. Effective and inclusive solutions to reduce disparities must consider factors, such as discrimination, trust, access to care, and quality of care, that can influence interactions between health care providers and women seeking diagnosis or treatment for an autoimmune disease or comorbid condition.

### 3.1. Clinical Guidelines

Clinical practice guidelines (CPGs) are evidence-based documents that carry the responsibility of outlining standards of care for all individuals. These fundamental resources in clinical practice have limited generalizability to diverse populations when there is a lack of scientific evidence, particularly for rare conditions or diverse subpopulations [49]. For example, various CPGs for lupus include clinical considerations for sex differences surrounding pregnancy and menopause; however, noticeably absent are diverse patient perspectives and guidance on the impact of race and ethnicity, social determinants of health, and holistic disease management on disease outcomes [50]. Effectiveness of CPGs is further hindered by a failure to include diverse perspectives in their development, including women and individuals from racial and ethnic minority groups or other disciplines, such as community health and epidemiology [51,52,53]. Guidelines developed through an intersectional lens, using representative data and multidisciplinary contributors have the potential to reduce disparities and improve outcomes for all women living with autoimmune disease.

Similar to practice guidelines, clinical interventions targeted for autoimmune diseases should address social determinants of health specific to the populations they aim to serve. It cannot be overstated that the road to improving guideline development and intervention implementation must contend with awareness and education gaps among health care providers regarding autoimmune diseases. When complaints about fatigue, changes in weight, sleep disturbances, or chronic pain are presented—typically to a primary care provider instead of a specialist—autoimmune diseases such as lupus or autoimmune thyroid diseases are too often misdiagnosed, dismissed, or not even considered. This can result in prolonged diagnostic times, delays in appropriate treatment, and worse treatment outcomes [3,48].

### 3.2. Patient–Provider Interactions

Effective implementation of clinical guidelines to eliminate health care disparities for women living with autoimmune diseases requires an assessment of patient–provider interactions. Two key elements for optimal patient–provider relationships are communication and trust. Both elements are essential to patient satisfaction, involvement in care, and adherence, and both parties need an unbiased understanding of how their identities can influence their communication style and the exchange of information.

According to the American College of Rheumatology, 59% of the adult rheumatology workforce is male, and less than 10% identify as belonging to a racial minority group, with Hispanic/Latinx having the largest representation (8.5%) [54,55]. Studies show that communication can be harmed by racial mismatch and gender discordance between providers and patients [56,57,58]. These influences can be heightened for women seeking medical care for autoimmune diseases, as gender can compound cultural differences in communication, especially in a field where only 41% of providers are the same sex as 80% of the patient population. Compared to men, African American women with lupus report more hurried and distracted communication, less compassion and respect, and more discrimination due to their race or ethnicity [59]. Racial mismatch among lupus patients has also resulted in fear of being stereotyped by their provider and less patient-centered decision making. For autoimmune conditions like lupus, where disease prevalence is highest among non-Hispanic Black women, differences in communication associated with racial and gender discordance with their primarily White and male providers has contributed to significant disparities in care delivery and outcomes [48,60,61]. Poor communication is linked with lower adherence to treatments and self-management, as well as reduced engagement with providers.

Communication can be further influenced by age, education level, health literacy, and assumed socioeconomic status, and when providers are unaware of or neglect these factors, disparities worsen, and women’s autoimmune health is further marginalized. Implicit bias and stereotypes can drive providers to withhold preventive care screening, treatment options, referrals to clinical trials, and referrals to specialists, resulting in unequal treatment of patients and confirming potential mistrust of the health care system [62]. Studies into lupus medication non-adherence show that some patients believe that their providers are withholding information or referrals, lack knowledge about their condition, and are unable to adequately explain treatments [61]. Similar experiences are reported with T1D patients, and their mistrust of providers can consequently influence the provider’s engagement in treatment [63]. Experiences with medical mistreatment or discrimination—which are well-documented among women and women of color—can shape how patients enter clinical interactions and how providers engage with their patients [64].

An intersectional solution for improving autoimmune patient–provider encounters should focus on addressing the impact of compounded identities on communication, trust, and trustworthiness between autoimmune disease clinicians and the heterogeneous population of women they serve. Other means to achieve women’s health equity include medical training or continuing education with a greater emphasis on patient-centered care, understanding biases and cultural sensitivity, and recruiting a diverse and multidisciplinary workforce that displays more concordance with the rising autoimmune patient population [65,66].

## 4. Patient-Centered Policy Solutions

Disparities in health care are both an outcome and a cause of multifaceted disadvantages and require inclusive policy solutions. Autoimmune diseases provide a robust case for complex influences at individual, interpersonal, community, and societal levels [67]. Given the influences highlighted in this review (e.g., sex, gender, race and ethnicity, life stage, education, lived experiences, etc.), combined with the whole-body impacts of autoimmune diseases and the life-long experience of managing a chronic condition that has multiple morbidities, disparities experienced by women living with autoimmune diseases are often compounded physically, financially, and psychosocially. The disadvantages often result in cyclical health outcomes that are very difficult to solve at the individual or community levels [56]. Thus, health care solutions in legislative, regulatory, and public policy become critical means to address the multi-level factors that perpetuate care disparities. These include policies that shape research funding, health care delivery, costs and coverage, and nondiscriminatory practices, particularly for vulnerable populations of women affected by autoimmune disease [68].

### 4.1. Access to Care

It is important to recognize the gradient of access to appropriate care for patients with autoimmune diseases; while patients may have easier access to primary care, their provider may not be familiar with their autoimmune disease, and patients may not have access to a specialist or experience long gaps between appointments. Limited access and education about preventative care, diagnosis processes, and innovative treatment options can also lead to delayed diagnoses, more advanced disease, and sometimes, irreversible health consequences [63]. For example, significant racial disparities have been documented in health care providers’ treatment of Black T1D patients regarding discussion, prescription, and use of diabetes technologies that have been shown to improve clinical outcomes [69].

Increasing access to care can also come from expanding the health care workforce by utilizing physician associates and assistants, community health workers, and nurses, as well as identifying innovative models to increase the quantity and distribution of providers with expertise in treating autoimmune diseases [55,65]. Strategies should include incentives for training medical professionals to specialize in rheumatology, as well as adjacent fields, such as endocrinology, nephrology, and gastroenterology, to meet the increasing autoimmune disease patient demand.

Research also shows that access to health care is worse for women who live in low-income households, lack quality or consistent employment, face racism or discrimination, and live in states with unfavorable healthcare policies [68]. Insurance coverage, differences in reimbursement, paucity of specialists, and distance from care all impact and are impacted by the financial health and stability of a patient. This is particularly relevant for women in United States, where three out of ten women are part of low-income households and one out of four women live in households with no workers. For women, having a low income or being uninsured results in greater barriers to quality care, poorly treated or untreated chronic conditions, higher morbidity, and reduced quality of life [64,70]. Therefore, addressing access and affordability gaps for women through regulatory policy and federal legislation is essential.

### 4.2. Health Care Coverage

Women with autoimmune diseases are at serious risk for financial toxicity, resulting from high disease burden, out-of-pocket (OOP) costs, challenges maintaining work productivity, and employment disability. In the United States, women pay USD 15 billion more each year in OOP costs than men, and the value of employer-sponsored coverage for women is USD 1.34 billion less than for men of the same age [71]. Even when insured, over 30% of women report that their plans do not always cover their necessary care or pay less than expected [68]. Utilization management practices like step therapy, which requires a patient to progress through a series of lesser treatment options before their insurance will cover a potentially optimal, but more expensive one, can result in unnecessary time and money spent by patients. Copays for specialists and medications can also become burdensome for a woman managing a chronic health condition like lupus or Hashimoto’s thyroiditis.

Policy solutions must revolutionize payment models for services to enhance coverage, reduce copays, and expand the clinical workforce for autoimmune disease care. New and innovative models for payment and reimbursement should be considered to better meet the needs of patients with autoimmune diseases. Expanding drug coverage through public insurance programs is particularly important for lower income patients who need assistance to manage a chronic health condition [65,68]. For T1D, diabetes-related costs total nearly USD 800 per month, with pharmacy costs accounting for over half of the monthly expenditures [72]. Women with T1D could take advantage of expanded coverage for outpatient care, prescriptions, supplies, and technology [63]. Policies should also address barriers to insurance eligibility and enrollment and moderate rising premium and OOP costs for patients [68].

Federal legislation that protects the financial health of individuals with chronic conditions is particularly helpful to women managing an autoimmune disease. The Help Ensure Lower Patient (HELP) Copays Act of 2023 (H.R. 830/S. 1375) will require health insurance plans to apply third-party payments, discounts, vouchers, and other reductions in out-of-pocket expenses towards the plan’s cost-sharing requirements [73]. This policy will allow patients to count external support towards reaching their OOP expense limits easier and sooner than if all the expenses required their direct contribution. The Safe Step Act of 2023 (H.R. 2630/S. 652) will help minimize delays and costs for autoimmune patients by its mandate for group health plans to establish exceptions to medication step therapy protocols and make the process clear and readily available to coverage recipients [74]. Additionally, state level policies—where private insurance is typically regulated—should also be intentional about protecting and expanding insurance coverage, including Medicaid programs, and safeguarding women with pre-existing conditions.

### 4.3. Funding Priorities

Much remains unknown about autoimmune diseases, including their exact causes, potential cures, and the full scope of their impact on women’s health. The National Institutes of Health Research Portfolio Online Reporting Tools system shares annual research investment data by condition or disease category. In 2022, NIH funded USD 1.079 billion in autoimmune disease research, approximately 2.4% of the federal agency’s budget. When these 2305 project titles were searched for a focus on women, only 32 project titles contained at least one of the following key words: sex, gender, female, maternal, or variations of women, pregnancy, or lactating. That same year, Congress recognized the need to coordinate efforts to address the increasing burden of autoimmune diseases on the U.S. population and their disproportionate impact on women and directed the NIH to establish an Office of Autoimmune Disease Research within the cross-cutting Office of Research on Women’s Health [75].

Funding agencies and entities must prioritize understanding autoimmune disease pathology, improving preventive care and diagnostics, and identifying new treatments and interventions for diverse subpopulations of women and across the lifespan. For interventions that prove effective at reducing disparities, long-term and sustainable funding of such efforts supports scaling up and maintaining these solutions for women’s health.

## 5. Future Directions

The disparities that have emerged for women with autoimmune diseases are undeniable, and they exist throughout every stage of the health care continuum—from bench to bedside to bureaucracy. Intersectionality provides the framework necessary to understand how and why different populations, like women, should be included and analyzed in a study, as well as how to implement interventions based on research discoveries and establish infrastructure and systems that ensure equity and access to quality health care solutions.

The diverse demographics of the U.S. population, including variations in sex, gender, age, health status, income, and geographic region, necessitate intentional inclusion in clinical studies to ensure generalizability and the correct interpretation of results. The long-term repercussion of not addressing the diverse patient perspectives of women with autoimmune diseases is the omission of patient needs and experiences that are essential to inform the interventions designed for their care. Increasing diverse populations of women in clinical trials is also key to understanding autoimmunity and disease mechanisms, identifying better diagnostics and treatments, and reducing health disparities and disease burden.

When Congress passed the NIH Revitalization Act of 1993, women and members of racial minority groups were required to be included as subjects in clinical research [76], yet, more than 30 years later, there is still much room for progress [27,77]. Sex and race are now mandated reporting metrics; however, the intersectional data on sex by race (or vice versa) is not [78]. With female patients making up the majority of autoimmune patients, it is critical to highlight subpopulations that may otherwise be excluded from clinical trials. Furthermore, with the movement toward precision medicine and applying machine learning models to research outcomes, women with autoimmune diseases must have holistic patient profiles incorporated into data analyses to support algorithms that can produce better outcomes. Perpetuating gaps in these datasets lead to inaccurate dosing guidelines, incomplete efficacy and safety data, and exacerbation of health disparities, resulting in a ripple effect of inadequate health care solutions that could continue to extend well beyond another 30 years.

Approaches to address disparities in autoimmune diseases must also consider the chronic, lifelong nature of many of these diseases which require significant medical intervention and self-management. A traditional care delivery model that places health care providers and patients as unequal partners should be re-evaluated to reduce inequalities and inequities, especially for chronic conditions like autoimmune diseases, which require greater patient participation in care. A clinical partnership that considers all of patients’ health identities and facilitates collaboration in decision-making and encourages a bidirectional flow of information, trust, and respect can yield greater patient participation, improve health outcomes, and reduce disparities [63]. While not a new concept, shared decision-making can be a particularly powerful technique that appreciates the unique experiences women with chronic autoimmune conditions face when seeking and communicating their care experience. Providers must create opportunities to increase patient self-efficacy in clinical encounters and consider the external factors which may impact women’s confidence and engagement in their health care. Promotion of self-efficacy enhances the rapport between a patient and a provider, and utilizing intersectionality frameworks in these clinical interactions can lead to patients feeling more understood and more likely to engage with the system, adhere to treatment, and exercise self-management.

Lastly, policies must strive to increase coverage for care models that support more comprehensive and complex care to address the evolving needs of autoimmune disease patients. State and federal policies can mitigate women’s health disparities in autoimmune diseases by encouraging and engaging scientific and community involvement in health care policy and advocacy. Notably, clinical guidelines have influenced funding for and access to testing and treatment innovations because they directly control supply and demand of resources for health care delivery [79]. Patients, clinicians, and researchers must be offered the opportunity to contribute their diverse experiences and expertise throughout the policy development process. As such, each perspective informs unique gaps and needs throughout the health care ecosystem that can be strategically assessed for sustainable and holistic policy solutions.

In conclusion, a comprehensive approach to address women’s health disparities in autoimmune diseases must incorporate an intersectional lens throughout, to ensure that medical research is intentional and inclusive; interventions are well-informed, appropriate, and culturally considerate; and decisions—both clinical and regulatory–are collaborative and have the capacity to meet the diverse and evolving needs of every woman.

## Figures and Tables

**Table 1:** Epidemiology and trends of systemic lupus erythematosus, Hashimoto’s thyroiditis, and type 1 diabetes mellitus in the United States.

Autoimmune Disease	Incidence	Prevalence	Female:Male	Race/Ethnicity Highlights
Systemic lupus erythematosus	4.77 per 100,000 population [18]	97.4 per 100,000 population [18]	9:1 [19]	**Female prevalence (per 100,000 person-years):** Black: 230.9; Hispanic: 120.7; White: 84.7; Asian/Pacific Islander: 84.4 **Male prevalence (per 100,000 person-years):** Black: 26.7; Hispanic: 18.0; Asian/Pacific Islander: 11.2; White: 8.9 [19]
Hashimoto’s thyroiditis	0.3–1.5 per 1000 population * [20]	11.7% of the U.S. population [21]	10:1 [22]	**Incidence rate (compared to White women):** Asian/Pacific Islander women: 31% decreased incidence; Black women: 33% decreased incidence [23]
Type 1 diabetes mellitus	22.2 per 100,000 persons [24]	5.3 per 1000 adults [25]	1:1.8 [26]	**Weighted prevalence (per 1000 adults):** Non-Hispanic White: 5.9; Non-Hispanic Black: 4.8; Hispanic: 4.0 [25]

* Global incidence rate.
